# The Role of I-FABP, REG3α, sCD14-ST, and LBP as Indicators of GI Tract Injury in MODS Patients

**DOI:** 10.3390/diagnostics15050515

**Published:** 2025-02-20

**Authors:** Yermek Turgunov, Alina Ogizbayeva, Sofiko Assamidanova, Dmitriy Matyushko, Miras Mugazov, Dana Amanova, Shynggys Nuraly, Yerzhan Sharapatov

**Affiliations:** 1 Department of Surgical Diseases, NJSC “Karaganda Medical University”, Karaganda 100008, Kazakhstan; turgunov@qmu.kz (Y.T.); grigorashvili@qmu.kz (S.A.); matyushko@qmu.kz (D.M.); amanovad@qmu.kz (D.A.); nurshintaurus@gmail.com (S.N.); 2 Department of Emergency Medical Care, Anaesthesiology and Resuscitation, NJSC “Karaganda Medical University”, Karaganda 100008, Kazakhstan; mugazov@kgmu.kz; 3 Department of Urology and Andrology, NJSC “Astana Medical University”, Astana 010000, Kazakhstan; erzhan.uro@gmail.com

**Keywords:** multiple organ dysfunction syndrome, MODS, bacterial translocation, intestinal permeability, GI tract

## Abstract

**Background/Objectives**: The aim of this study was to evaluate potential biomarkers of bacterial translocation (lipopolysaccharide-binding protein (LBP) and soluble CD14 subtype (sCD14-ST)) and intestinal wall damage (intestinal fatty acid binding protein (I-FABP), Zonulin, and regenerating islet-derived protein-3α (REG3α)) in patients with multiple organ dysfunction syndrome (MODS). **Methods**: The study involved 327 patients divided into two groups: Group 1 comprised 227 patients with MODS (main group), while Group 2 comprised 100 patients with identical pathologies but without MODS (control group). To examine these biomarkers in the blood, venous blood was taken in the control group on the day of admission to the hospital, in patients with MODS on the first day of MODS staging, and later on Days 3 and 7 of its development. Levels of these markers in blood serum were determined by enzyme-linked immunosorbent assays according to the manufacturers’ instructions. **Results:** In the control group, values of all the investigated markers were lower than in the group of MODS patients (*p* < 0.0001). In the main group, the mortality rate was 44.9% (*n* = 102). The values of sCD14-ST on Day 1 and of I-FABP and REG3α on Days 1 and 3 were higher in deceased MODS patients (*p* < 0.05), while LBP levels on Day 7 were conversely lower in the deceased patients (*p* = 0.006). SOFA and APACHE II scores were higher in the deceased patients (*p* < 0.0001). **Conclusions:** In MODS patients, the increased I-FABP, REG3α, and sCD14-ST but decreased LBP levels may indicate increased intestinal wall permeability and bacterial translocation, which may exacerbate the course of multiple organ dysfunction and increase the risk of mortality. Despite the limitations of this study, the studied potential biomarkers can be considered noteworthy candidates for identifying MODS patients at high risk of mortality.

## 1. Introduction

Multiple organ dysfunction syndrome (MODS) is characterized by the dysfunction of two or more organs that occurs simultaneously. These dysfunctions mainly occur in the cardiovascular system, the respiratory system, and in renal, hepatic, neurological, and coagulation disorders. MODS often occurs in intensive care unit (ICU) patients. Due to the development of MODS, patients may stay in the ICU longer, and in severe conditions, the risk of death tends to increase to up to 100% [1]. ICU patients are heterogeneous, and they are admitted with different and often unrelated conditions, but for many of them, MODS is a common pathogenetic pathway leading to death.

The mechanisms and pathogenesis of MODS development are currently the subject of discussion. However, it has been suggested that intestinal disorders play a major role in the development of MODS [2]. Many factors in ICU patients in severe/critical conditions can lead to intestinal disorders: systemic inflammatory response syndrome (SIRS), which is characterized by overwhelming immune responses that lead to free radical generation; hypoperfusion of the intestinal mucosa, causing hypoxia, which releases reactive oxygen and nitrogen species; intestinal peristalsis disorders; insufficient enteral nutrition; and the proliferation of pathogenic nosocomial bacteria [3,4]. Microcirculatory disorders of the intestinal mucosa lead to hypoperfusion, mucosal edema, mucosal ischemia, and an increase in free oxygen radicals that destroy the mucosal cytoskeleton. These changes contribute to the disruption of the integrity of the intestinal barrier, which increases the intestinal permeability and facilitates the entry of pathogens into extraluminal spaces and mesenteric lymph nodes that results in migration of gut microorganisms to distant sites (bacterial translocation) [5,6]. By entering the bloodstream, bacteria and their endotoxins increase the systemic inflammatory response, increasing the risk of MODS, sepsis, and mortality [5,7].

Assuming that MODS patients exhibit significant changes in biomarkers of intestinal wall damage and bacterial translocation, the aim of this study was to evaluate these biomarkers in MODS patients. I-FABP (intestinal fatty acid binding protein), REG3α (regenerating islet-derived protein-3α), and Zonulin were selected as potential markers of intestinal wall damage. As potential markers of bacterial translocation, sCD14-ST (soluble CD14 subtype; presepsin) and LBP (LPS-binding protein; lipopolysaccharide-binding protein) were selected.

I-FABP is an cytosolic enterocytic protein that plays a role in intracellular fatty acid transport [8]. Low levels of I-FABP can be detected in serum due to the constant release of mature enterocytes as part of normal intestinal homeostasis, whereas when enterocytes are damaged, I-FABP levels increase significantly [9].

REG3α is an antimicrobial lectin C-peptide produced and secreted by epithelial cells of the small intestine into the intestinal lumen [10]. REG3α also helps maintain the intestinal barrier function by reducing intestinal epithelial cell apoptosis, and it also reduces oxidative stress and inflammatory responses in intestinal epithelial cells. When the intestinal barrier is damaged, REG3α penetrates the epithelium, translocates to the lamina propria, and subsequently enters the systemic circulation [11,12].

Zonulin is a protein that plays a significant role in intestinal wall permeability. It opens tight epithelial junctions between duodenal and jejunal cells, which leads to increased intestinal wall permeability. To open tight junctions, Zonulin activates the epidermal growth factor receptor via proteinase-activated receptor 2. Activation of these two receptors reduces the transepithelial electrical resistance, which leads to increased intestinal wall permeability. Therefore, serum Zonulin is considered a biomarker of tight junction stability and paracellular barrier integrity [13].

There are two states of CD14 receptors: a membrane-bound form on macrophages and monocytes (mCD14) and a soluble form that circulates in the bloodstream (sCD14). The binding of mCD-14 to various components of microorganisms via Toll-like receptors (TLRs) triggers proinflammatory pathways with the production of cytokines (interleukin-1, interleukin-6, interleukin-8, tumor necrosis factor) and the activation of phagocytosis of bacterial pathogens. As a result, mCD14 undergoes proteolysis to form sCD14-ST, which can be detected in the bloodstream within 1.5 to 2 h after the onset of infection [14,15].

LBP is an acute-phase protein produced in response to bacteremia. It specifically interacts with lipid A of bacterial lipopolysaccharide (LPS). The LPS-LBP complex then binds to the CD14 receptor and promotes a proinflammatory response with the release of cytokines. Due to its long half-life (2–3 days), LBP levels are detectable in serum for a long time after bacteremia, and it is a reliable marker of bacterial translocation [16].

MODS is a critical condition that requires extensive clinical management and huge healthcare resources. Therefore, identifying the potential prognostic markers in MODS patients at high risk of death can be beneficial for decreasing mortality.

## 2. Materials and Methods

### 2.1. Study Design

**Figure 1:**
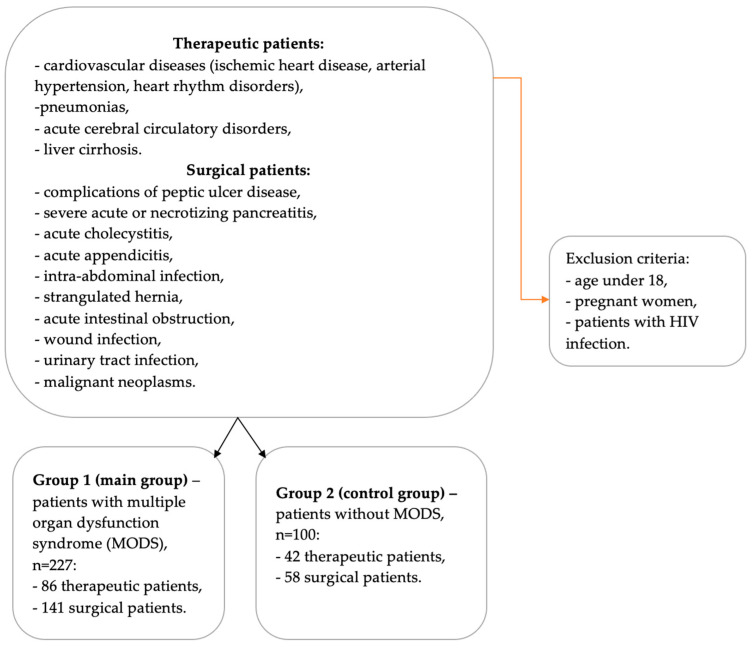
Schematic representation of the inclusion and exclusion criteria for the study patients.

The study was conducted within the period from July 2023 to August 2024 at the facilities of 4 hospitals in the city of Karaganda: Regional Clinical Hospital, Multidisciplinary Hospitals No. 1 and No. 3, and the Medical University Clinic. The study involved 327 patients divided into 2 groups: Group 1—227 patients with MODS (main group); and Group 2—100 patients with identical pathologies but without MODS (control group). Patients under 18 years of age, pregnant women, and patients with HIV infection were excluded (Figure 1).

MODS was assessed by using the SOFA (Sequential Organ Failure Assessment) scale [17], and the mortality rate was calculated by using the APACHE II (Acute Physiology and Chronic Health Evaluation II) scale [18].

To determine the levels of the biomarkers in blood, venous blood was drawn from the control group on the day of admission to the hospital. In the MODS patients, venous blood was drawn on the first day of MODS staging and, later, on Days 3 and 7 of its development.

Venous blood was collected in 5 mL test tubes with a coagulation activator and a serum gel separator. Test tubes with blood were placed in a refrigerator (4–8 °C) until transportation from the hospital to the laboratory. Transportation was carried out in a strictly vertical position in a transport container with ice (2–8 °C). Then, the blood samples were centrifuged for 20 min at 1000× *g*, after which the gel completely separated the serum from the clot, forming a tight barrier. The obtained sample of freshly prepared serum was stored for up to 2 months at –20 °C to –80 °C to avoid loss of biological activity and contamination. Repeated freeze/thaw cycles were not allowed.

The levels of sCD14-ST, LBP, I-FABP, REG3α, and Zonulin in blood serum were determined by enzyme-linked immunosorbent assays (ELISAs) using the ELISA robotic system Evolis from BioRad (Hercules, CA, USA), using commercial kits for each of the studied markers according to the manufacturer’s instructions (Cloud-Clone Corp., Katy, TX, USA). The microplate provided in each kit was precoated with biotinylated antibodies specific for that marker. Standards and patient serum samples were added to each well of the microplate and incubated at 37 °C for 1 h. Then avidin conjugated with horseradish peroxidase was added to each well and incubated at 37 °C. After the addition of the tetramethylbenzidine substrate solution, only those wells containing the specific marker changed color, and these changes were measured spectrophotometrically at a wavelength of 450± 10 nm. The level of each marker in the samples was then determined by comparing the absorbance of the samples with a standard calibration sample [19].

### 2.2. Trial Registration

This study is registered on ClinicalTrials.gov. The research registration unique identifying number (UIN) is NCT06221293. URL: https://clinicaltrials.gov/study/NCT06221293 (last update posted 19 April 2024).

### 2.3. Statistical Methods

For statistical processing, STATISTICA 8.0 (StatSoft) was used. For each indicator, the median (Me) and the lower and upper quartiles (Q1–Q3) were calculated. As for the qualitative signs, the proportion and frequency of occurrence was calculated. The non-parametric Mann–Whitney criterion for quantitative variables and the χ^2^-square criterion and the exact Fisher’s criterion for qualitative variables were used. The diagnostic capacity of the biomarker levels was evaluated by a receiver operating characteristic (ROC) analysis, and the optimal cut-offs were obtained with the Youden Index. The results were considered significant at *p* < 0.05.

## 3. Results

### 3.1. Patients

The control and main groups did not differ in age, sex, and comorbid pathology (*p* = 0.133, *p* = 0.672, and *p* = 0.441, respectively; refer to Table 1 below). The control group patients had identical pathologies to the MODS patients (*p* = 0.582). Therapeutic patients (86 in the main group and 42 in the control group) were admitted with the following diagnoses: cardiovascular diseases (ischemic heart disease, arterial hypertension, heart rhythm disorders), pneumonias, acute cerebral circulatory disorders, and liver cirrhosis. Surgical pathologies (141 in the main group and 58 in the control group) included complications of peptic ulcer disease, severe acute or necrotizing pancreatitis, acute cholecystitis, acute appendicitis, intra-abdominal infection, strangulated hernia, acute intestinal obstruction, wound infection, urinary tract infection, and malignant neoplasms.

### 3.2. Biomarkers of Intestinal Wall Damage and Bacterial Translocation in the Studied Groups

**Figure 2:**
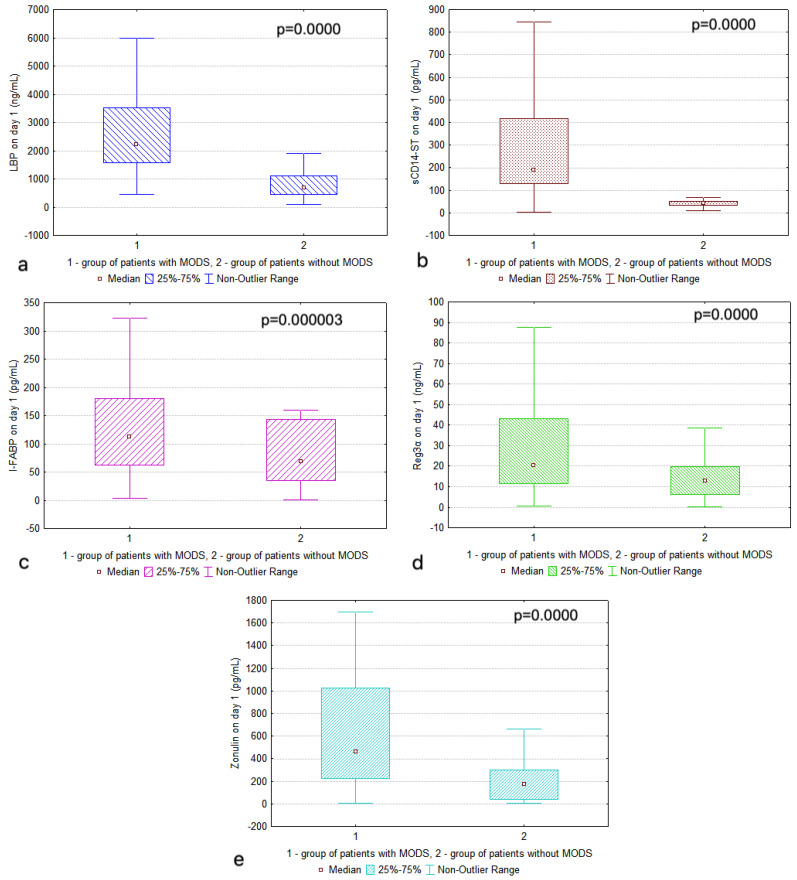
The levels of (**a**) lipopolysaccharide-binding protein (LBP), (**b**) soluble CD14 subtype (sCD14-ST), (**c**) intestinal fatty acid binding protein (I-FABP), (**d**) regenerating islet-derived protein-3α (REG3α), and (**e**) Zonulin on Day 1 in the study groups. The Mann–Whitney statistical test was used to identify significant differences for all markers between the study groups.

In the control group, the values of all the studied markers were significantly lower than in the group of patients with MODS (refer to Table 2 and Figure 2). In terms of dynamics (marker comparison on Days 1, 3, and 7), no changes in any of the markers were found in the main group (*p* = 0.081 for LBP; *p* = 0.525 for sCD14-ST; *p* = 0.862 for I-FABP; *p* = 0.538 for Reg3α; *p* = 0.111 for Zonulin).

### 3.3. Biomarkers of Intestinal Wall Damage and Bacterial Translocation in MODS Patients in Terms of Mortality

**Figure 3:**
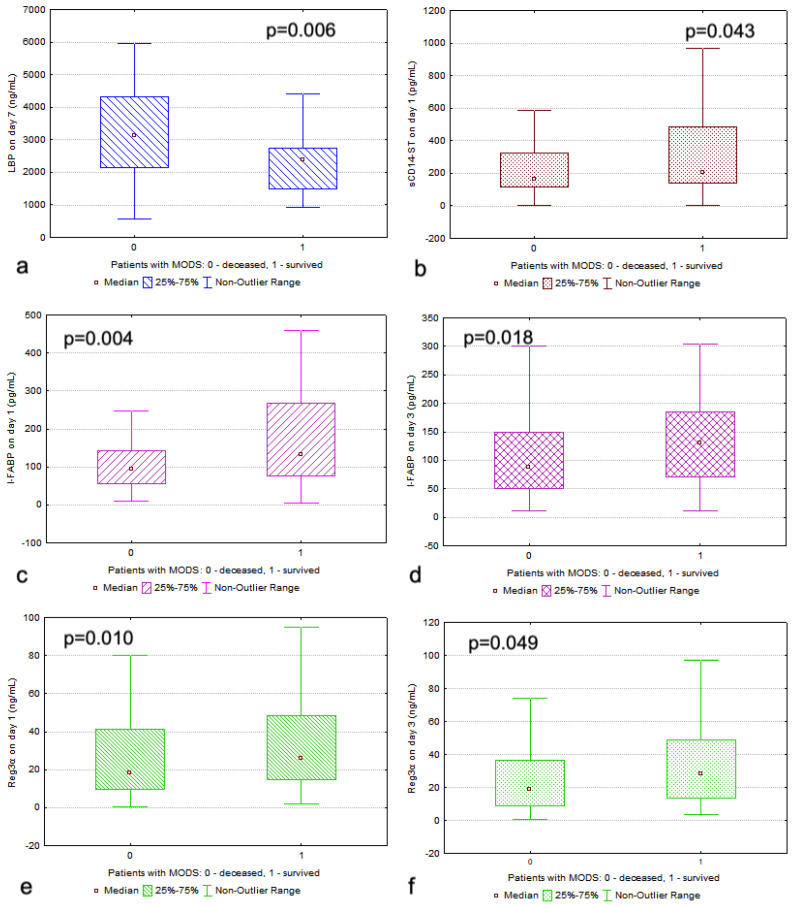
The levels of (**a**) lipopolysaccharide-binding protein (LBP), (**b**) soluble CD14 subtype (sCD14-ST), (**c**,**d**) intestinal fatty acid binding protein (I-FABP), and (**e**,**f**) regenerating islet-derived protein-3α (REG3α) in deceased and surviving patients with multiple organ dysfunction syndrome (MODS). The Mann–Whitney statistical test was used to identify significant differences for all markers between the study groups.

In deceased patients, sCD14-ST on the first day and I-FABP and REG3α on the first and third days were significantly higher (*p* = 0.043, *p* = 0.004, *p* = 0.018, *p* = 0.010, and *p* = 0.049, respectively; refer to Table 3 and Figure 2). The LBP levels on the seventh day were conversely lower in the deceased patients (*p* = 0.006; Table 3 and Figure 3). As expected, the SOFA and APACHE II scores were significantly higher in the deceased patients (*p* < 0.0001).

### 3.4. ROC Analysis of Studied Markers in MODS Patients for the Prediction of Mortality

**Figure 4:**
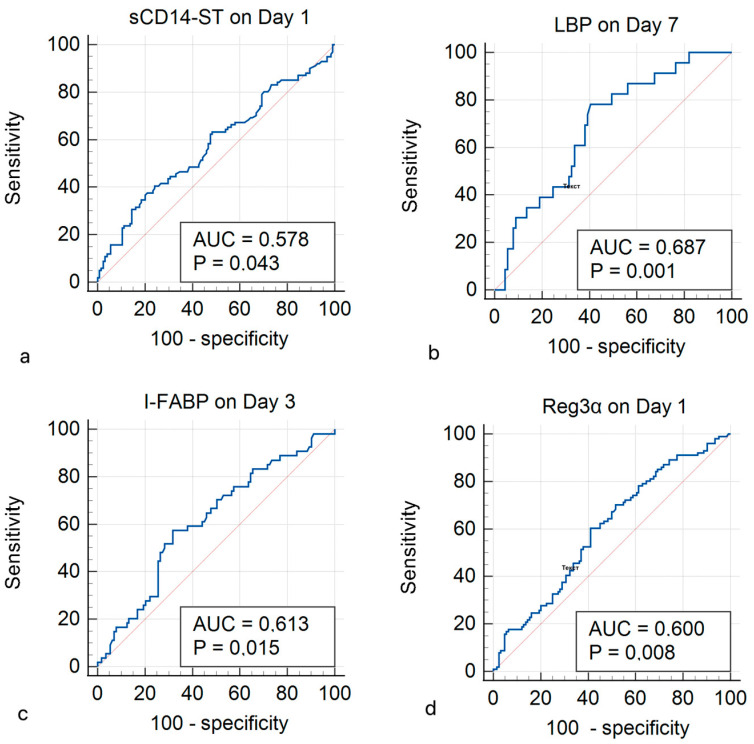
ROC analysis of the (**a**) soluble CD14 subtype (sCD14-ST), (**b**) lipopolysaccharide-binding protein (LBP), (**c**) intestinal fatty acid binding protein (I-FABP), and (**d**) regenerating islet-derived protein-3α (REG3α) in multiple organ dysfunction syndrome patients for the prediction of mortality.

The ROC analysis was performed to determine the threshold values of the studied markers at which the risk of mortality in patients with MODS increases. The results are presented in Table 4 and Figure 4 below. Although statistically significant differences between the deceased and surviving MODS patients were obtained for these markers, the sensitivity/specificity results were quite low for some of the markers.

## 4. Discussion

Patients in intensive care units are heterogeneous, as they are admitted with various, and often unrelated, conditions. However, many studies on ICU patient in severe/critical conditions showed that MODS was the primary cause of death. Determining the risk factors for mortality in MODS may help in risk stratification and initiating early intensive care measures. To date, gastrointestinal disorders have played a central role in triggering the cascade of events leading to the development of MODS [1,20]. The intestinal barrier is formed by mucus, intestinal epithelial cells, and immune cells. All this prevents bacteria from penetrating the systemic bloodstream. ICU patients in severe conditions develop changes in various body systems, including the intestine. The centralization of blood circulation due to splanchnic vasoconstriction leads to hypoxia and ischemia of the intestinal wall [21]. The permeability of the intestinal wall increases; as a result, bacteria and/or their endotoxins from the intestine penetrate the systemic bloodstream (bacterial translocation) and further enhance the immune response, which becomes systemic, ultimately leading to multiple organ dysfunction [5]. Late recognition of intestinal wall damage increases the risk of worsening multiorgan dysfunction, the development of sepsis, acute respiratory distress syndrome, and mortality. The maintenance of normal intestinal mucosal function is very important to prevent intestinal barrier dysfunction and gut bacterial translocation [22].

sCD14-ST and LBP are considered the most suitable potential biomarkers of bacterial translocation. The mCD-14 membrane protein binds to various bacterial components via Toll-like receptors and triggers a proinflammatory immune response, forming soluble sCD14-ST, which is detected in the systemic bloodstream [8]. LBP is produced by hepatocytes in response to bacteremia and pathogen-associated molecular patterns (PAMPs). LBP bind them to immune system cells, which subsequently causes the release of cytokines like TNF-α, IL-1, IL-6, and IL-12 [23]. Therefore, sCD14-ST and LBP are considered potential markers of bacterial translocation and the development of septic complications [8,23,24].

Several studies have shown that sCD14-ST levels are a predictor of sepsis, adverse outcomes, and 28-day mortality (threshold 685 pg/mL), and they correlate with changes in SOFA and APACHE II scores [20,25,26,27,28]. Previous studies have shown that there are significant differences in sCD14-ST levels in patients with SIRS and sepsis. In the study by Vodnik et al., in patients with SIRS, the sCD14-ST levels ranged between 170 and 689 pg/mL, while in the sepsis patients, these values were 639–4223 pg/mL [29]. In the study by Juroš et al., the mean sCD14-ST level in patients without sepsis was 525.5 pg/mL, while in patients with sepsis, it was 1121.5 pg/mL [15]. In Zhang et al.’s meta-analysis of 11 studies, the sCD14-ST threshold ranged from 317 to 729 pg/mL (sensitivity 0.70–1.00; specificity 0.62–0.93) [27]. This study confirmed that the sCD14-ST level was higher on Day 1 in the group of MODS patients (191.62 pg/mL) compared with the control group (41.90 pg/mL), and it was also higher in deceased MODS patients. The threshold value of sCD14-ST, above which signified an increased risk of mortality, was determined to be >378 pg/mL, which was within the threshold values found in previous studies, although the sensitivity was quite low (37.62%).

According to various studies, serum LBP levels were significantly higher at admission in patients with SIRS, sepsis, and septic shock compared with a group of healthy subjects and in non-survivors compared with survivors. But over the time, they gradually decreased; a decrease in LBP levels was observed in 71.3% of survivors and in 38.5% of non-survivors [30,31]. In this study, it was also confirmed that LBP levels were higher in the MODS patient group (2231.95 ng/mL) compared with that in the control group (684.76 ng/mL). However, it was found that deceased MODS patients had a significantly lower LBP level on Day 7 (2379.75 ng/mL) than did surviving patients (3118.85 ng/mL). The threshold value of LBP was defined as ≤2727.55 ng/mL, but the specificity of this result was only 59.55%. The results obtained can be explained by data from other studies: higher concentrations of LBP have a protective effect against bacterial infection and may represent a physiological defense mechanism against infection. Some authors suggested that higher LBP levels in the acute phase of systemic inflammation can inhibit the binding of bacterial LPS to immune cells in the blood circulation, thereby reducing the production of cytokines [32]. For example, in mice with bacteremia, intraperitoneal administration of LBP prevented hepatic failure and reduced the mortality rate. The authors of that study are convinced that high concentrations of LBP have a protective effect against microbial infection [33]. It was also found that critically ill ICU patients with less elevated LBP levels had significantly worse outcomes, which was explained by the fact that the patients with rapidly progressive sepsis could not adequately synthesize LBP, thus failing to adequately respond to any systemic microbial infection [31,33]. These results may support the hypothesis that MODS patients with a more unfavorable course and a higher risk of mortality have changes in their sCD14-ST and LBP levels, suggests increased bacterial translocation in this category of patients, which would aggravate the course of multiple organ dysfunction syndrome.

In addition to identifying the biomarkers of bacterial translocation of the intestinal microflora, it is important to identify the biomarkers of intestinal wall damage, since it is the increased permeability of the intestinal mucosa that leads to the increased bacterial translocation. In various studies, I-FABP, REG3α, and Zonulin have shown to be potential markers of intestinal wall damage. I-FABP is present in the cytoplasm of enterocytes, and its detection in plasma indicates enterocyte membrane disruption, with levels above 100 pg/mL indicating mesenteric ischemia and enterocyte necrosis [34]. There is evidence that I-FABP, as a marker, is very sensitive, since it can be detected at an early stage of small bowel ischemia, even when the histological damage is minor [35]. I-FABP levels correlated with disease severity and clinical outcomes in patients with necrotizing enterocolitis, mesenteric ischemia, hemorrhagic shock, cardiogenic shock, HIV infection, COVID-19, and septic shock, as well as in critically ill ICU patients [11,36,37,38,39,40]. High plasma I-FABP levels were associated with higher mortality, which correlated with the SOFA and APACHE II scores [39,40]. In this study, it was found that I-FABP levels were significantly higher in the MODS patient group (112.00 pg/mL) compared with the control group (68.48 pg/mL). I-FABP levels were significantly higher in the deceased MODS patients (130.50 pg/mL) than in the surviving patients (88.30 pg/mL). The threshold value of I-FABP was defined as >118.2 pg/mL, which does not contradict previous studies, even though the sensitivity of this result was only 57.41%.

Zonulin is able to open intracellular tight junctions between enterocytes by actin polymerization via protein kinase C. Thus, the higher the concentration of Zonulin, the more the number of tight junctions that are opened and the higher the permeability of the intestinal wall [13]. Zonulin increases in the blood serum of patients with celiac disease, inflammatory bowel disease, obesity, diabetes mellitus, HIV, COVID-19, and sepsis (regardless of the genesis of sepsis, abdominal and non-abdominal) [11,41,42]. Zonulin in serum was elevated in the sepsis patients and the deceased patients, which also correlated with the SOFA and APACHE II scores [39,43]. The threshold value of serum Zonulin for predicting mortality was >1.5 ng/mL [44]. In this study, it was found that Zonulin levels were significantly higher in the MODS patient group (458.90 pg/mL) compared with the control group (173.63 pg/mL). Although Zonulin levels were not significantly higher in the deceased MODS patients (493.84 pg/mL) than in the survivors (416.10 pg/mL), no statistical significance was found (*p* > 0.05).

REG3α is secreted by Paneth cells into the intestinal lumen; it maintains the intestinal barrier by reducing inflammatory reactions in the epithelium and participates in antibacterial defense. When the intestinal permeability increases, REG3α is released from the intestinal lumen into the systemic bloodstream [45]. This marker is rather new and understudied, but it has already shown potential as a marker of intestinal wall damage as it correlates with sCD14 and LBP levels, as well as with levels of lipopolysaccharide, IL-6 and IL-8 [12,13,46]. In this study, it was found that REG3α levels were significantly higher in the MODS patient group (20.40 ng/mL) compared with the control group (12.90 ng/mL). Deceased MODS patients had significantly higher REG3α levels (25.97 ng/mL) than surviving patients (18.53 ng/mL). The threshold value of REG3α was defined as >20.4 ng/mL, but the specificity of this result was only 58.87%.

The results obtained may support the hypothesis that due to impaired intestinal microcirculation, MODS patients in severe/critical conditions experience a cascade of reactions that disrupts the intestinal protective barrier against pathogens (as evidenced by the increased levels of I-FABP, REG3α, and Zonulin in the blood). These changes lead to increased bacterial translocation, resulting in an enhanced systemic immune response, a worsened course of organ dysfunction, and an increased risk of mortality. The presented markers of intestinal damage could potentially be diagnostic criteria for intestinal dysfunction in addition to the existing multiple organ dysfunction scales, such as SOFA.

A potential limitation of this study may be the heterogeneity of patients selected by the underlying disease that led to the development of MODS (the sample included both therapeutic and surgical patients with different pathologies), which may have resulted in the low sensitivity and specificity of the ROC analysis; further studies in this direction are required.

## 5. Conclusions

In MODS patients, the increased I-FABP, REG3α, and sCD14-ST and decreased serum LBP levels may indicate increased intestinal wall permeability and increased bacterial translocation, which may exacerbate the course of multiple organ dysfunction and increase the risk of mortality. Despite the limitations of this study, the potential biomarkers of intestinal wall damage and bacterial translocation studied can be considered noteworthy candidates for identifying patients at high risk of mortality in order to revise their therapy to reduce their ICU stay time and mortality.

## Figures and Tables

**Table 1:** Characteristics of patients in the main and control groups.

Criterion/Group		Patients with MODS (*n* = 227)	Patients Without MODS (*n* = 100)	*p*-Level
Age		64.0 (51.0–73.0)	62.0 (46.5–70.0)	0.133
Sex	Male	51.5%	49.0%	0.672
	Female	48.5%	51.0%	
Comorbidities	+	78.9%	75.0%	0.441
	−	21.1%	25.0%	
Main Pathology	Medical	38.0%	42.0%	0.582
	Surgical	62.0%	58.0%	
SOFA scores (Day 1)		5.0 (3.0–8.0)	0.0 (0.0–0.0)	**0.0000**
APACHE II scores (Day 1)		16.0 (11.0–21.0)	6.0 (4.0–8.0)	**0.0000**
Mortality	+	44.9%	0%	**0.0000**
	−	55.1%	100%	

Notes: for the age criterion, SOFA and APACHE II scores, the median (Me) and lower and upper quartiles (Q1–Q3) are given.

**Table 2:** The lipopolysaccharide-binding protein (LBP), soluble CD14 subtype (sCD14-ST), intestinal fatty acid binding protein (I-FABP), regenerating islet-derived protein-3α (REG3α), Zonulin levels on Day 1 in both study groups.

Criterion	Patients with MODS (*n* = 227)	Patients Without MODS (*n* = 100)	Z	*p*-Level
LBP (ng/mL)	2231.95 (1569.30–3513.25)	684.76 (451.38–1106.70)	10.827	**0.0000**
sCD14-ST (pg/mL)	191.62 (128.47–416.20)	41.90 (32.95–50.47)	13.122	**0.0000**
I-FABP (pg/mL)	112.00 (63.00–180.80)	68.48 (35.06–143.12)	4.708	**0.000003**
Reg3α (ng/mL)	20.40 (11.55–43.04)	12.90 (6.36–19.66)	5.710	**0.0000**
Zonulin (pg/mL)	458.90 (222.25–1023.33)	173.63 (38.70–297.79)	8.006	**0.0000**

Notes: The median (Me) and lower and upper quartiles (Q1–Q3) are given. Z: Mann–Whitney criteria values.

**Table 3:** The LBP, sCD14-S, I-FABP, REG3α, and Zonulin levels and the SOFA and APACHE II scores on Day 1, Day 3, and Day 7 after the MODS diagnosis in terms of mortality.

Criterion		Mortality − (*n* = 125)	Mortality + (*n* = 102)	*p*-Level
LBP (ng/mL)	Day 1	2256.68 (1431.65–3407.70)	2230.70 (1630.60–3809.25)	0.686
	Day 3	2535.55 (1600.20–3890.05)	2008.03 (1528.20–3267.75)	0.166
	Day 7	3118.85 (2156.20–4318.75)	2379.75 (1489.45–2727.55)	**0.006**
sCD14-ST (pg/mL)	Day 1	165.98 (118.60–322.32)	204.24 (137.65–483.46)	**0.043**
	Day 3	171.29 (118.60–345.87)	202.20 (124.46–455.20)	0.232
	Day 7	165.15 (111.40–365.77)	235.20 (107.06–393.16)	0.503
I-FABP (pg/mL)	Day 1	93.70 (57.10–143.30)	131.30 (76.20–267.10)	**0.004**
	Day 3	88.30 (50.00–150.10)	130.50 (71.00–185.30)	**0.018**
	Day 7	86.80 (50.40–126.60)	101.50 (54.00–140.00)	0.342
Reg3α (ng/mL)	Day 1	18.53 (9.62–41.30)	25.97 (14.96–48.25)	**0.010**
	Day 3	18.62 (9.10–36.67)	28.16 (13.57–48.90)	**0.049**
	Day 7	16.57 (8.78–31.80)	22.04 (11.16–52.22)	0.131
Zonulin (pg/mL)	Day 1	416.10 (215.62–917.84)	493.84 (228.62–1243.33)	0.276
	Day 3	453.80 (217.58–876.40)	511.24 (244.92–1213.33)	0.238
	Day 7	437.68 (224.44–931.88)	599.54 (258.80–727.79)	0.732
SOFA scores	Day 1	4.0 (3.0–5.0)	7.0 (5.0–9.0)	**0.0000**
	Day 3	3.0 (2.0–5.0)	7.0 (5.0–8.0)	**0.0000**
	Day 7	2.0 (0.5–3.0)	6.0 (4.0–10.0)	**0.0000**
APACHE II scores	Day 1	12.0 (9.0–16.0)	20.0 (16.0–24.0)	**0.0000**
	Day 3	9.5 (6.0–14.0)	20.0 (13.5–23.0)	**0.0000**
	Day 7	8.0 (5.0–12.0)	16.5 (12.0–27.0)	**0.00002**

Notes: Median (Me) and lower and upper quartiles (Q1–Q3) are given.

**Table 4:** ROC analysis of the studied markers in MODS patients for the prediction of mortality.

ROC Analysis Results	AUC (95% CI)	*p*-Level	Youden J-Index	Optimal Threshold Value	Sensitivity	Specificity
sCD14-ST on Day 1	0.578 (0.511–0.644)	**0.043**	0.167	>378	37.62	79.03
LBP on Day 7	0.687 (0.592–0.771)	**0.001**	0.378	≤2727.55	78.26	59.55
I-FABP on Day 3	0.613 (0.535–0.687)	**0.015**	0.256	>118.2	57.41	68.14
Reg3α on Day 1	0.600 (0.533–0.665)	**0.008**	0.193	>20.4	60.40	58.87

Notes: AUC (95% CI) is the area under the ROC curve, where 95% CI is the confidence interval and the *p*-level is the significance level.

## Data Availability

The data generated in this study are available upon request from the corresponding author.

## Abbreviations

The following abbreviations are used in this manuscript:

MODS	Multiple organ dysfunction syndrome
GI	Gastrointestinal
ICU	Intensive care unit
I-FABP	Intestinal fatty acid binding protein
REG3α	Regenerating islet-derived protein-3α
mCD14	Membrane CD14
sCD14-ST	Soluble CD14 subtype
TLR	Toll-like receptors
LBP	Lipopolysaccharide-binding protein
LPS	Lipopolysaccharide
SIRS	Systemic inflammatory response syndrome
SOFA	Sequential Organ Failure Assessment
APACHE II	Acute Physiology and Chronic Health Evaluation II
ELISA	Enzyme-linked immunosorbent assay
UIN	Unique identifying number
URL	Uniform Resource Locator
Me	Median
Q1–Q3	Lower and upper quartiles
ROC	Receiver operating characteristic
AUC	Area under the curve
CI	Confidence interval
IL	Interleukin
TNF	Tumor necrosis factor
